# Endopelvic Fascia Reconstruction During Robotic Assisted Laparoscopic Radical Prostatectomy: A Novel Robotic Surgical Technique

**DOI:** 10.51894/001c.143927

**Published:** 2025-09-30

**Authors:** Mohamed Abdelhady

## 21

### INTRODUCTION

Stress urinary incontinence is one of the main side effects following robotic assisted laparoscopic radical prostatectomy (RALP). Multiple surgical techniques have been proposed to improve urinary incontinence following RALP including: Retzius sparing, hood technique, posterior reconstruction (Rocco stitch), and anterior reconstruction (Patel stitch).

### HYPOTHESIS

We are presenting our technique for complete endopelvic fascia reconstruction following RALP and evaluating the continence rates for our patients following the usage of this new surgical technique.

### METHODS

A retrospective analysis was performed by reviewing the charts of the patient’s undergoing RALP from January 1st 2022 to December 13th 2023 after obtaining IRB approval. For all these patient’s, the endopelvic fascia reconstruction technique was utilized. After completion of the vesicourethral anastomosis, a 3-0 V-lock suture is used to re-approximate the puboprostatic ligaments to the serosal layer of the bladder bilaterally. Continence was evaluated at 1-month, 3-month, 6-month and 12-month intervals. Continence was defined as no leakage of urine and no diapers or pads for over a 24-hour period.

### RESULTS

A total of 66 patients were evaluated. 6 were excluded. Of the excluded patients, 2 were lost to follow-up, 2 had concomitant urge incontinence, 1 had a urethral disruption after a trauma and 1 developed a bladder neck contracture. At the 1-month period, 18.33% of patients were continent. At the 3- and 6-month visits, the continence rates improved to 58.33% and 88.33%, respectively. At 1 year, all but 1 of the patients was continent, a continence rate of 98.33%.

### CONCLUSION

Traditional RALP continence rates prior to advanced reconstruction ranged from 60%-80% at 6 months and improved to 84%-87% at 1 year. With our endopelvic fascia reconstruction technique, we had an improvement in continence rates compared to traditional technique continence rates.

